# Clinicopathological Features of Endoscopically Resected Early-Onset Colorectal Neoplasia Compared with Later-Onset Cases

**DOI:** 10.3390/cancers18030509

**Published:** 2026-02-04

**Authors:** Naohiko Akimoto, Shun Nakagome, Ryosuke Inoue, Rina Motomiya, Yuka Shimazu, Tsugumi Habu, Eriko Koizumi, Kazutoshi Higuchi, Takayoshi Nishimoto, Jun Omori, Ryuji Ohashi, Atsushi Tatsuguchi, Katsuhiko Iwakiri, Masanori Atsukawa

**Affiliations:** 1 Department of Gastroenterology, Graduate School of Medicine, Nippon Medical School, Tokyo 113-8603, Japan; 2 Department of Integrated Diagnostic Pathology, Nippon Medical School, Tokyo 113-8603, Japan

**Keywords:** advanced adenoma, advanced neoplasia, colonoscopy, early-onset colorectal cancer, endoscopic resection, pedunculated, young-onset

## Abstract

**Background:** The incidence of colorectal cancer diagnosed before age 50 has been increasing worldwide. However, limited data describe the endoscopic and pathological features of colorectal lesions encountered and treated during routine colonoscopy in younger adults. This study aimed to characterize age-related differences in endoscopically resected colorectal neoplasia. **Methods:** We conducted a retrospective, single-center observational study of consecutively endoscopically resected colorectal neoplasia at a high-volume academic teaching hospital in Japan. Patient-level and lesion-level characteristics were compared between early-onset (<50 years) and later-onset (≥50 years) groups. Lesions were evaluated for location, morphology, size, histology, resection method, and advanced neoplasia status. **Results:** A total of 1299 patients with 3399 lesions were analyzed, including 498 early-onset patients with 940 lesions. Early-onset neoplasia showed a left-sided predominance, with higher proportions in the distal colon and rectum. Pedunculated morphology was more frequently observed in early-onset lesions. Early-onset disease was also associated with larger lesion size, a higher prevalence of high-grade tubular adenoma, and increased rates of advanced adenoma and advanced neoplasia, resulting in more frequent use of endoscopic mucosal resection or submucosal dissection. **Conclusions:** Endoscopically resected colorectal lesions in younger adults exhibit distinct anatomical and morphological features compared with later-onset cases, indicating heterogeneity at the premalignant stage.

## Simple Summary

Colorectal cancer occurring in adults younger than 50 years has been increasing worldwide, but little is known about the characteristics of the early colorectal lesions that are actually found and removed during routine colonoscopy in this age group. In this study, we examined more than 3000 colorectal lesions removed endoscopically at a large academic hospital and compared their features between younger and older patients. We found that lesions in younger adults were more often located in the left side of the colon, were more likely to have a stalked (pedunculated) shape, and more frequently showed advanced pathological features. These findings suggest that colorectal lesions in younger adults may differ from those in older individuals even before cancer develops. A better understanding of these differences may help improve future research and guide endoscopic practice for younger patients.

## 1. Introduction

The incidence of colorectal cancer diagnosed before fifty years has been increasing across multiple countries during the past several decades, while rates in older adults have stabilized or declined in many settings, a pattern synthesized in recent cancer statistics and comparative registry analyses [1,2,3,4,5,6,7]. This epidemiologic shift contributed to the decision in the United States to lower the starting age for average-risk screening to forty-five [8]. Accumulating evidence suggests that individuals aged forty-five to forty-nine have non-trivial prevalence of any neoplasia and advanced neoplasia that approaches values observed in those aged fifty to fifty-nine [9,10], suggesting the crucial role of screening colonoscopy for young individuals. In younger patients, colorectal lesions exhibit unique clinicopathological features [11] and are more commonly located in the distal colon and rectum, consistent with early-onset colorectal cancer [3,12,13,14,15,16]. These observations underscore the need to characterize with precision the earliest lesions that endoscopists actually encounter and remove in routine practice among younger adults. Despite the accelerating literature on early-onset colorectal cancer, much of the current evidence arises from population screening cohorts and cancer registries rather than from systematically profiled lesions resected endoscopically at an early stage [2,3,17]. This gap is clinically important because decisions at the time of colonoscopy depend on subsite, macroscopic morphology, size and histology, features that can vary by age at diagnosis and that may influence the selection of resection methods and subsequent surveillance [18,19]. Despite the growing literature on early-onset colorectal cancer, limited data are available on the endoscopic phenotypes of lesions actually encountered and treated in routine practice, particularly in younger adults. A clearer understanding of age-related differences in lesion location, morphology, size, and histology is needed to inform real-time endoscopic decision-making. This study focuses specifically on lesions removed endoscopically in routine colonoscopy and compares patient-level and lesion-level characteristics by age, aiming to generate directly actionable evidence for endoscopic management in younger adults.

## 2. Methods

### 2.1. Study Design

We conducted a retrospective, single-center observational study of consecutively endoscopically resected colorectal neoplasia at Nippon Medical School Hospital (Tokyo, Japan) during the study period from 1 February 2011 to 31 December 2021.

The study protocol was approved by the Institutional Review Board of Nippon Medical School (No. M-2025-339).

### 2.2. Participants: Eligibility and Exclusions

All colorectal adenomas and carcinomas resected endoscopically by polypectomy, endoscopic mucosal resection, or endoscopic submucosal dissection during the study period were included, provided that both endoscopic and histopathology reports were available. Both lesion-level and patient-level datasets were constructed, corresponding to per-lesion and per-patient analyses. The exclusion criteria included non-neoplastic lesions, hereditary colorectal cancer syndromes such as Lynch syndrome and familial adenomatous polyposis, and inflammatory bowel disease. For patients who underwent colonoscopy before and after the age of 50 years, only the initial endoscopic treatment was included in the analysis.

### 2.3. Definitions and Variable Coding

**Figure 1:**
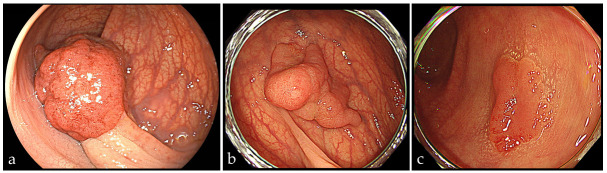
Endoscopically resected lesions classified according to the Paris classification as (**a**) pedunculated type (Ip), (**b**) sessile type (Is), and (**c**) superficial elevated type (IIa).

Early-onset defined as diagnosis < 50 years, later-onset as ≥50 years, consistent with previous early-onset colorectal cancer studies. Anatomical location was documented using six colonic subsites (cecum, ascending colon, transverse colon, descending colon, sigmoid colon, and rectum). For secondary analyses, lesions were additionally grouped into three anatomical regions—proximal colon, distal colon, and rectum—according to standard segmental classification. Macroscopic morphology was determined based on the Paris classification and categorized as Ip, Is/Isp, or type II. In addition, a dichotomous variable for pedunculated morphology was created, classifying lesions as pedunculated (Ip) (Figure 1) versus non-pedunculated (non-Ip). Histopathological diagnosis was classified as carcinoma, tubular adenoma with low-grade dysplasia, tubular adenoma with high-grade dysplasia, or serrated lesions, including sessile serrated lesion and traditional serrated adenoma/serrated adenoma, in accordance with the 2019 World Health Organization Classification of Tumors of the Digestive System (5th edition) [20] and Japanese Society for Cancer of the Colon and Rectum criteria [21]. Advanced adenoma was defined as a lesion ≥10 mm, with villous/tubulovillous histology, or with high-grade dysplasia. In Japan, lesions diagnosed pathologically as “intramucosal carcinoma (pTis)” are considered equivalent to high-grade dysplasia in Western terminology, and were therefore included as advanced adenoma. Advanced neoplasia was defined as advanced adenoma or pT1 carcinoma.

### 2.4. Data Sources and Collection

We abstracted demographic, lifestyle (smoking, alcohol), family history of colorectal cancer, endoscopic, and histopathological data from the institutional endoscopy reporting system and pathology database using a standardized case report form. Variables and categories correspond to those summarized in the baseline and lesion.

### 2.5. Statistical Analysis

Chi-square test or Fisher exact test was used for categorical variables and Mann–Whitney U test for continuous variables. Fisher’s exact test was applied when the expected cell count was <5 in any cell.

Lesion-level multivariable analysis was performed to identify factors independently associated with pedunculated morphology. Because multiple lesions could originate from the same patient, generalized estimating equations (GEE) with a binomial distribution and logit link function were used to account for within-patient correlation. Patient identifier was specified as the clustering variable, assuming an exchangeable working correlation structure, and robust (empirical) standard errors were applied. The dependent variable was pedunculated morphology (Ip vs. non-Ip). The multivariable model included onset age (<50 vs. ≥50 years), lesion size (<10 mm vs. ≥10 mm), sex, location, histologic category, family history of colorectal cancer, smoking status, and alcohol consumption as covariates. Adjusted odds ratios (ORs) with 95% confidence intervals (CIs) were estimated.

To address potential selection and indication bias related to colonoscopy indication, we performed a sensitivity analysis restricted to colorectal neoplasia detected by screening or surveillance colonoscopy and resected endoscopically.

Statistical significance was defined as a two-sided *p* value < 0.05. All analyses were performed using SPSS Statistics, version 27 (IBM Corp., Armonk, NY, USA).

## 3. Results

We analyzed 1299 patients and 3399 lesions. The early-onset group included 498 patients and 940 lesions. The later-onset group included 801 patients and 2459 lesions.

Baseline characteristics showed that sex and body mass index did not differ between groups, whereas family history status and exposure behaviors showed clear between-group differences with substantial unknown categories in both groups (Table 1).

Regarding the location of colorectal neoplasia using three-region schema, distal colon (50% vs. 33%) and rectum (10% vs. 7.9%) were higher in early-onset (*p* < 0.001) (Table 2). The same left-shift appeared in the adenoma subset (*p* < 0.001) (Table 3). As for macroscopic morphology, Is or Isp was the most frequent morphology in both age groups (64% for early-onset vs. 73% for later-onset) (Table 2). The pedunculated type was markedly more common in early-onset with a significant difference (25% vs. 6.7%; *p* < 0.001) (Table 2). These patterns were reproduced in the adenoma subset (23% vs. 5.7%; *p* < 0.001) (Table 3). Across both age groups, the most frequently observed size category was 5–9 mm (49% for early-onset, 48% for later-onset neoplasia, respectively); however, early-onset neoplasia displayed a greater proportion of lesions measuring at least 10 mm (31% for early-onset vs. 22% for later-onset, respectively) and a reduced proportion of lesions smaller than 4 mm (20%), and this pattern was recapitulated in the adenoma-only subset (26% and 17%, respectively (*p* < 0.001)) (Table 3). Histopathological profiles varied by age at diagnosis. Early-onset disease showed a higher proportion of high-grade tubular adenoma than later-onset disease, and this difference was evident both in analyses of all colorectal neoplasia and in the adenoma-only subset (Table 2 and Table 3). Regarding advanced lesions, the prevalence of advanced neoplasia (39% vs. 27%; *p* < 0.001) and adenoma (34% vs. 22%; *p* < 0.001) were higher in early-onset compared with later onset (Table 2 and Table 3). Compared with later-onset cases, early-onset cases showed differences in resection methods: cold snare resection was performed less frequently (24% vs. 55%), whereas endoscopic mucosal resection or endoscopic submucosal dissection was performed more often (76% vs. 45%) (Table 2 and Table 3). When limited to advanced adenomas, location and morphology showed trends similar to those observed in colorectal neoplasia (*p* < 0.001, respectively), and pedunculated types were significantly more frequent in early-onset advanced adenomas compared with later-onset counterparts (44% vs. 15%; *p* < 0.001) (Supplementary Table S1). In an analysis restricted to pedunculated-type (Ip) lesions, no significant differences were observed between early- and later-onset groups regarding clinicopathologic features. However, within this Ip-only cohort, early-onset cases showed a lower proportion of lesions < 4 mm compared with later-onset cases (0.9% vs. 5.5%) (Supplementary Table S2). Sensitivity analyses that used both six-site and six age categories yielded consistent results (Supplementary Table S3).

In lesion-level multivariable analysis using generalized estimating equations, early-onset status (<50 years) was independently associated with a higher likelihood of pedunculated morphology (adjusted OR 5.52, 95% CI 3.42–8.90; *p* < 0.001). Lesion size showed a strong dose–response relationship, with lesions measuring ≥ 10 mm having markedly higher odds of pedunculated (Ip) morphology compared with lesions < 10 mm (*p* < 0.001). No significant associations were observed for sex, location, histology, family history, smoking status, or alcohol consumption (Table 4).

In a sensitivity analysis restricted to colorectal neoplasia detected by screening or surveillance colonoscopy, early-onset neoplasia showed a different distribution of location compared with later-onset neoplasia, with a lower proportion of proximal lesions and a higher proportion of distal and rectal lesions (*p* = 0.012 for three-site classification). In contrast, lesion size distribution, histologic composition, and the prevalence of advanced neoplasia did not significantly differ between early- and later-onset groups (Supplementary Table S4).

## 4. Discussion

We set out to delineate age-related differences in early-onset colorectal lesions actually managed in routine practice, focusing on neoplasia that were detected and removed endoscopically. In this consecutively accrued cohort, early-onset disease showed a consistent left-sided predilection across the three-region schema, with higher proportions in the distal colon and rectum and a lower proportion in the proximal colon than later-onset disease. Macroscopic morphology was dominated by Is or Isp in both age groups, yet pedunculated lesions were distinctly more frequent in early-onset, indicating a tangible shift in gross architecture with clinical relevance for snare selection and resection planning. Histologically, early-onset exhibited a higher proportion of high-grade tubular adenoma, with corresponding increases in advanced adenoma and advanced neoplasia, reinforcing the impression that biologically and procedurally meaningful differences emerge well before cancer diagnosis.

In the present study, early-onset colorectal lesions treated by endoscopic resection demonstrated a significantly higher proportion of advanced adenoma compared with later-onset counterparts. Notably, this enrichment was accompanied by an association with pedunculated morphology, suggesting that macroscopic growth patterns may partially underlie the clinicopathologic features of early-onset premalignant neoplasia. Although advanced adenomas in younger individuals have traditionally been considered uncommon, accumulating evidence indicates that early-onset adenoma represents a biologically and clinically relevant entity rather than a benign incidental finding [22].

Several population-based and cohort studies have shown that individuals diagnosed with early-onset adenoma, particularly advanced adenoma, experience a measurable risk of subsequent advanced neoplasia or colorectal cancer, emphasizing the importance of accurate risk stratification in this age group [23,24]. Furthermore, advanced adenomas detected in very young patients are frequently sporadic rather than syndromic, underscoring the need for careful lesion-level phenotyping beyond hereditary cancer screening alone [25]. Otherwise, surveillance studies following removal of high-risk adenomas have also demonstrated that younger patients do not necessarily exhibit a lower risk of metachronous advanced neoplasia than older patients, challenging the assumption that age alone confers biological indolence [26,27].

In parallel, epidemiologic investigations have identified lifestyle and dietary factors associated with early-onset advanced or high-risk adenomas, including lesions with high-grade dysplasia, supporting the concept that distinct exposure profiles may contribute to early tumorigenesis [28,29]. Large-scale screening and meta-analytic studies further indicate that advanced premalignant lesions constitute a substantial proportion of early-age onset colorectal neoplasia detected in average-risk populations, reinforcing the clinical relevance of this disease spectrum [9].

Importantly, studies focusing on T1 early-onset colorectal cancer have reported a higher likelihood of lymph node metastasis compared with later-onset disease, suggesting that biological aggressiveness may already be established at the earliest invasive stages [30]. In this context, precise characterization of advanced adenomas in younger patients—particularly with respect to macroscopic morphology—may be critical for identifying lesions at higher risk of progression. While prior studies have examined risk factors or registry-based outcomes, relatively few investigations have leveraged large-scale databases constructed through rigorous verification of both endoscopic appearance and histopathologic diagnosis. The present study addresses this gap by integrating detailed endoscopic morphology with pathology-confirmed lesion data, thereby providing a more robust framework for understanding early-onset advanced colorectal neoplasia.

Pedunculated colorectal lesions including both adenomas and early carcinomas are defined by clear endoscopic and morphological criteria using the Paris classification [31], and usually larger than 1 cm and occur most frequently in the sigmoid, rectosigmoid, or descending colon, although cases appear in the ascending colon, cecum, and transverse colon as well. To our knowledge, this is the first report showing that the proportion of pedunculated types have been more common in early-onset neoplasia, particularly adenomas, when compared with the later-onset group. Our findings are consistent with previous observational results describing the proportion of pedunculated types in young cohorts. Comparing with previous observational findings describing the proportion of pedunculated types in young cohorts, previous report indicated that lesion size and pedunculated morphology were independent factors for advanced adenomas [32]. Conversely, a retrospective study showed that the incidence of sessile polyps in the proximal colon increases with age [33].

Although all detected lesions are ultimately removed, our findings have several clinically implications beyond simple resection. First, the higher prevalence of pedunculated morphology in early-onset lesions has practical relevance during real-time colonoscopy, as lesion morphology directly influences snare selection, electrocautery use, and the anticipation of procedure-related adverse events. Second, the increased frequency of advanced histologic features among early-onset lesions suggests that post-polypectomy risk stratification and surveillance planning may warrant particular attention in this population, even at the premalignant stage. Finally, characterization of distinct endoscopic phenotypes before cancer development provides etiologic insight into early-onset colorectal neoplasia, supporting the hypothesis that early-onset disease may follow different growth patterns from later-onset disease. Together, these findings underscore the relevance of lesion-level phenotyping in early-onset colorectal neoplasia, even in the context of universal endoscopic removal.

A key interpretative issue raised by this study is the potential influence of colonoscopy indication across age groups. Younger patients more commonly undergo colonoscopy because of symptoms such as hematochezia or abdominal pain, or following a positive fecal occult blood test, thereby constituting an “enriched” high-risk population. In contrast, later-onset patients include a substantial proportion of asymptomatic individuals undergoing routine screening or surveillance. Under such circumstances, apparent differences in lesion size or advanced histologic features could reflect differences in baseline risk profiles rather than intrinsic age-related biological differences. To address this concern, we conducted a sensitivity analysis restricted to colorectal neoplasia detected by screening or surveillance colonoscopy and resected endoscopically. Within this restricted cohort, age-related differences in location persisted, whereas lesion size, histologic features, and the prevalence of advanced neoplasia were comparable between early- and later-onset groups. Because all lesions in this sensitivity analysis were endoscopically resected, the number of eligible early-onset cases was necessarily limited, and firm conclusions cannot be drawn from this subset alone. Nevertheless, these findings suggest that at least some of the observed age-related patterns—particularly in lesion distribution—are not solely attributable to symptomatic case enrichment and may reflect differences in disease presentation at the premalignant stage.

This study has several limitations. First, this was a single-center study, which may limit generalizability. However, the study was conducted at a high-volume academic teaching hospital in Japan, where standardized, high-quality endoscopic and pathological practices are routinely implemented, supporting the internal validity of the findings. Second, the analysis was restricted to endoscopically resected lesions, and therefore does not allow estimation of the true prevalence of diminutive or unresected colorectal lesions. Nonetheless, our institutional policy favors endoscopic removal of lesions considered clinically relevant, and the present cohort predominantly represents lesions ≥ 5 mm, which are of greater clinical significance in routine practice. Third, interobserver consistency of the Paris classification was not formally quantified in this study; however, endoscopic morphology was routinely reviewed and confirmed by multiple endoscopists in a teaching-hospital setting, which may have mitigated individual subjectivity. Fourth, a proportion of patients had missing information on family history of colorectal cancer and lifestyle-related variables, including, smoking status and alcohol consumption. In particular, family history is a well-established risk factor for colorectal neoplasia, and the presence of unknown data limited our ability to fully evaluate its association with lesion characteristics. However, the primary objective of this study was to characterize endoscopically resected lesion-level phenotypes, and the observed age-related differences in anatomical distribution and macroscopic morphology are unlikely to be solely explained by these unmeasured lifestyle factors. Finally, the study population consisted almost exclusively of Japanese and Asian patients, raising the possibility of limited applicability to other ethnic groups. Further studies in more diverse populations will be necessary to confirm the generalizability of these findings.

This study has several notable strengths. First, it was conducted at a high-volume academic teaching hospital, where colonoscopy is performed either by experienced expert endoscopists or by well-trained trainees under close supervision. All endoscopic assessments made by trainees are routinely reviewed and confirmed by multiple senior endoscopists, ensuring a consistently high diagnostic standard. Second, histopathological evaluation was independently performed by at least two board-certified pathologists, which minimizes interpretive bias and ensures adherence to standardized diagnostic criteria. Third, based on these clinically and pathologically validated specimens, we established a meticulously reviewed, lesion-level database encompassing more than 1000 patients and over 3000 lesions. This curated resource includes comprehensive clinicopathological annotation with minimal missing data, enabling robust and reliable analyses. Finally, our institutional practice is to endoscopically resect almost all lesions considered clinically relevant. Accordingly, this study focuses on lesions of genuine clinical importance, rather than diminutive findings with limited impact, providing results that are directly applicable to real-world endoscopic practice.

## 5. Conclusions

Colorectal neoplasia detected and removed endoscopically in younger adults demonstrated distinct clinicopathological features, including a left-sided distribution, a higher prevalence of pedunculated morphology, and an enrichment of advanced adenomas, compared with later-onset disease. These findings highlight that age-related differences are already evident at the premalignant stage and have direct implications for real-time endoscopic decision-making, including resection strategy and post-polypectomy surveillance. Future multicenter and multiethnic studies integrating molecular and environmental data will be essential to clarify the biological mechanisms underlying these phenotypic differences and to refine risk-adapted endoscopic management in younger populations.

## Figures and Tables

**Table 1:** Baseline characteristics of patients with early- and later-onset colorectal neoplasia.

	Total (n = 1299)	Patients with Early-Onset Neoplasia (n = 498)	Patients with Later-Onset Neoplasia (n = 801)	*p* Value
Age				
median (IQR)	59 (46–73)	44 (41–47)	71 (62–77)	<0.001
Sex				0.73
Female, n (%)	410 (32%)	160 (32%)	250 (31%)	
Male, n (%)	889 (68%)	338 (68%)	551 (69%)	
Body mass index				
Median (IQR)	23 (21–26)	23 (21–26)	24 (21–26)	0.84
Body mass index (categorized)				0.27
<25 kg/m^2^, n (%)	861 (66%)	321 (65%)	540 (67%)	
≥25 kg/m^2^, n (%)	438 (34%)	177 (35%)	261 (33%)	
Family history of colorectal cancer				<0.001
Absent, n (%)	504 (39%)	253 (51%)	251 (31%)	
Present, n (%)	92 (7.1%)	49 (9.8%)	43 (5.4%)	
Unknown, n (%)	703 (54%)	196 (39%)	507 (63%)	
Smoking habit				<0.001
Never, n (%)	523 (40%)	192 (39%)	331 (41%)	
Past, n (%)	192 (15%)	55 (11%)	137 (17%)	
Current, n (%)	313 (24%)	165 (33%)	148 (19%)	
Unknown, n (%)	271 (21%)	86 (17%)	185 (23%)	
Alcohol consumption				<0.001
Non-drinker, n (%)	507 (39%)	140 (28%)	367 (46%)	
1–4 times/week, n (%)	284 (22%)	144 (29%)	140 (17%)	
≥5 times/week, n (%)	339 (26%)	147 (29%)	192 (24%)	
Unknown, n (%)	169 (13%)	67 (14%)	102 (13%)	
Indications for colonoscopy				<0.001
Screening/surveillance, n (%)	312 (24%)	51 (10%)	261 (32%)	
Therapeutic resection, n (%)	987 (76%)	447 (90%)	540 (67%)	

Abbreviation: IQR, interquartile range.

**Table 2:** Clinicopathological characteristics of early- vs. later-onset colorectal neoplasia resected endoscopically.

	Total (n = 3399)	Early-Onset Neoplasia (n = 940)	Later-Onset Neoplasia (n = 2459)	*p* Value
Location (6 sites)				<0.001
Cecum, n (%)	226 (6.6%)	43 (4.6%)	183 (7.4%)	
Ascending, n (%)	792 (23%)	147 (16%)	645 (26%)	
Transverse, n (%)	808 (24%)	184 (20%)	624 (25%)	
Descending, n (%)	323 (9.5%)	92 (9.8%)	231 (9.4%)	
Sigmoid, n (%)	957 (28%)	376 (40%)	581 (24%)	
Rectum, n (%)	293 (8.6%)	98 (10%)	195 (7.9%)	
Location (3 sites)				<0.001
Proximal, n (%)	1826 (54%)	374 (40%)	1452 (59%)	
Distal, n (%)	1280 (38%)	468 (50%)	812 (33%)	
Rectum, n (%)	293 (8.6%)	98 (10%)	195 (7.9%)	
Morphology				<0.001
Ip, n (%)	396 (12%)	232 (25%)	164 (6.7%)	
Is or Isp, n (%)	2411 (71%)	603 (64%)	1808 (73%)	
II, n (%)	592 (17%)	105 (11%)	487 (20%)	
Pedunculated type				<0.001
Ip, n (%)	396 (12%)	232 (25%)	164 (6.7%)	
Non-Ip, n (%)	3003 (88%)	708 (75%)	2295 (93%)	
Lesion size (mm)				<0.001
Median (IQR)	6 (4–9)	7 (5–10)	6 (4–8)	
Lesion size (categorized)				<0.001
<4 mm, n (%)	926 (27%)	190 (20%)	736 (30%)	
5–9 mm, n (%)	1640 (48%)	460 (49%)	1180 (48%)	
≥10 mm, n (%)	833 (25%)	290 (31%)	543 (22%)	
Histology				<0.001
Carcinoma (pTis, pT1), n (%)	277 (8.1%)	90 (9.6%)	187 (7.6%)	
high-grade TA, n (%)	436 (13%)	172 (18%)/	264 (11%)	
low-grade TA, n (%)	2544 (75%)	645 (69%)	1899 (77%)	
SSL, TSA, SA, n (%)	142 (4.2%)	33 (3.5%)	109 (4.4%)	
Advanced neoplasia, n (%)	1031 (30%)	364 (39%)	667 (27%)	<0.001
Endoscopic resection methods				<0.001
Cold polypectomy, n (%)	1590 (47%)	229 (24%)	1361 (55%)	
Hot snare polypectomy/EMR/ESD, n (%)	1809 (53%)	711 (76%)	1098 (45%)	

Abbreviations: EMR, Endoscopic mucosal resection; ESD, Endoscopic submucosal dissection; IQR, interquartile range; SSL, sessile serrated lesion; TSA, traditional serrated adenoma; TA, tubular adenoma.

**Table 3:** Clinicopathological characteristics of early- vs. later-onset colorectal adenoma resected endoscopically.

	Total (n = 3122)	Early-Onset Adenomas (n = 850)	Later-Onset Adenomas (n = 2272)	*p* Value
Location (6 sites)				<0.001
Cecum, n (%)	213 (6.8%)	39 (4.6%)	174 (7.7%)	
Ascending, n (%)	752 (24%)	143 (17%)	609 (27%)	
Transverse, n (%)	772 (25%)	171 (20%)	601 (27%)	
Descending, n (%)	299 (9.6%)	85 (10%)	214 (9.4%)	
Sigmoid, n (%)	849 (27%)	331 (39%)	518 (23%)	
Rectum, n (%)	237 (7.6%)	81 (10%)	156 (6.9%)	
Location (3 sites)				<0.001
Proximal, n (%)	1737 (56%)	353 (41%)	1384 (61%)	
Distal, n (%)	1148 (37%)	416 (49%)	732 (32%)	
Rectum, n (%)	237 (7.6%)	81 (9.5%)	156 (6.9%)	
Morphology				<0.001
Ip, n (%)	325 (10%)	196 (23%)	129 (5.7%)	
Is or Isp, n (%)	2294 (74%)	566 (67%)	1728 (76%)	
II, n (%)	503 (16%)	88 (10%)	415 (18%)	
Pedunculated type				<0.001
Ip, n (%)	325 (10%)	196 (23%)	129 (5.7%)	
Non-Ip, n (%)	2797 (90%)	654 (77%)	2143 (94%)	
Lesion size (mm)				
Median (IQR)	6 (4–9)	7 (5–10)	6 (4–8)	
Lesion size				<0.001
<4 mm, n (%)	925 (30%)	189 (22%)	736 (32%)	
5–9 mm, n (%)	1605 (51%)	444 (52%)	1161 (51%)	
≥10 mm, n (%)	592 (19%)	217 (26%)	375 (17%)	
Histology				<0.001
high-grade TA, n (%)	436 (14%)	172 (20%)	264 (11%)	
low-grade TA, n (%)	2544 (82%)	645 (76%)	1899 (84%)	
SSL, TSA, SA, n (%)	142 (4.5%)	33 (3.9%)	109 (4.8%)	
Advanced adenoma, n (%)	792 (25%)	291 (34%)	501 (22%)	<0.001

Abbreviations: IQR, interquartile range; SSL, sessile serrated lesion; TSA, traditional serrated adenoma; TA, tubular adenoma.

**Table 4:** Multivariable lesion-level analysis for pedunculated (Ip) morphology.

Variable	Adjusted OR	95% CI	*p* Value
Onset age			<0.001
<50	5.52	3.42–8.90	
≥50	1 (reference)		
Lesion size			<0.001
≥10 mm	7.36	4.45–12.2	
<10 mm	1 (reference)		

Patient identifier was specified as the clustering variable, assuming an exchangeable working correlation structure, and robust (empirical) standard errors were applied. The dependent variable was pedunculated morphology (Ip vs. non-Ip). The multivariable model included onset age (<50 vs. ≥50 years), lesion size (<10 mm vs. ≥10 mm), sex, location, histologic category, family history of colorectal cancer, smoking status, and alcohol consumption as covariates. Abbreviations; CI, confidence interval; OR, odds ratio.

## Data Availability

The data presented in this study are available from the corresponding author upon reasonable request, due to ethical and privacy restrictions.

## Supplementary Materials

The following supporting information can be downloaded at https://www.mdpi.com/article/10.3390/cancers18030509/s1: Table S1: Clinicopathological characteristics of early- vs. later-onset colorectal advanced adenoma resected endoscopically; Table S2: Clinicopathological characteristics of early- vs. later-onset pedunculated type colorectal neoplasia resected endoscopically; Table S3: Clinicopathological characteristics of colorectal neoplasia resected endoscopically according to age at diagnosis; Table S4: Clinicopathological characteristics of early- vs. later-onset colorectal neoplasia detected by screening or surveillance colonoscopy and resected endoscopically.
