# Supplementary material for: Clinicopathological Features of Endoscopically Resected Early-Onset Colorectal Neoplasia Compared with Later-Onset Cases
Source: Cancers (Basel). 2026 Feb 4;18(3):509. doi: 10.3390/cancers18030509 (PMC12897112; doi:10.3390/cancers18030509)
Supplement: Supplementary file 1 [file cancers-18-00509-s001.zip › cancers-4102624-supplementary.pdf]

Supplementary Table S1. Clinicopathological characteristics of early- vs later-onset colorectal advanced adenoma resected endoscopically.

	**Early-onset adenomas (n = 364)**	**Later-onset adenomas (n = 667)**	***P* value**
Location (6 sites)			<0.001
Cecum, n (%)	19 (5.2 %)	48 (7.2 %)	
Ascending, n (%)	51 (14 %)	171 (26 %)	
Transverse, n (%)	58 (16 %)	125 (19 %)	
Descending, n (%)	31 (8.5 %)	51 (7.6 %)	
Sigmoid, n (%)	164 (45 %)	189 (28 %)	
Rectum, n (%)	41 (11 %)	83 (12 %)	
Location (3 sites)			<0.001
Proximal, n (%)	128 (35 %)	344 (52 %)	
Distal, n (%)	195 (54 %)	240 (36 %)	
Rectum, n (%)	41 (11 %)	83 (12 %)	
Morphology			<0.001
Ip, n (%)	161 (44 %)	102 (15 %)	
Is or Isp, n (%)	137 (38 %)	359 (54 %)	
II, n (%)	66 (18 %)	206 (31 %)	
Pedunculated type			<0.001
Ip, n (%)	161 (44 %)	102 (15 %)	
Non-Ip, n (%)	203 (56 %)	565 (85 %)	
Lesion size			0.59
<4 mm, n (%)	15 (4.1 %)	20 (3.0 %)	
5-9 mm, n (%)	71 (20 %)	125 (19 %)	
≥10 mm, n (%)	278 (76 %)	522 (78 %)	
Histology			0.068
Carcinoma (pTis, pT1), n (%)	73 (20 %)	166 (25 %)	
high-grade TA, n (%)	172 (47 %)	264 (40 %)	
low-grade TA, n (%)	100 (28 %)	189 (28 %)	
SSL, TSA, SA, n (%)	19 (5.2 %)	48 (7.2 %)	

Abbreviations; SSL, sessile serrated lesion; TSA, traditional serrated adenoma; TA, tubular adenoma.

Supplementary Table S2. Clinicopathological characteristics of early- vs later-onset pedunculated type colorectal neoplasia resected endoscopically.

	**Early-onset adenomas (n = 232)**	**Later-onset adenomas (n = 164)**	***P* value**
Location (6 sites)			0.50
Cecum, n (%)	0 (0 %)	0 (0 %)	
Ascending, n (%)	40 (17 %)	30 (18 %)	
Transverse, n (%)	35 (15 %)	31 (19 %)	
Descending, n (%)	9 (3.9 %)	10 (6.1 %)	
Sigmoid, n (%)	138 (60 %)	84 (51 %)	
Rectum, n (%)	10 (4.3 %)	9 (5.5 %)	
Location (3 sites)			0.47
Proximal, n (%)	75 (32 %)	61 (37 %)	
Distal, n (%)	147 (63 %)	94 (57 %)	
Rectum, n (%)	10 (4.3 %)	9 (5.5 %)	
Lesion size			0.021
<4 mm, n (%)	2 (0.9 %)	9 (5.5 %)	
5-9 mm, n (%)	85 (37 %)	55 (34 %)	
≥10 mm, n (%)	145 (62 %)	100 (61 %)	
Histology			0.085
Carcinoma (pTis, pT1), n (%)	36 (16 %)	35 (21 %)	
High-grade TA, n (%)	72 (31 %)	37 (23 %)	
Low-grade TA, n (%)	114 (49 %)	89 (54 %)	
Serrated adenoma, n (%)	10 (4.3 %)	3 (1.8 %)	
Advanced neoplasia, n (%)	161 (69 %)	102 (62 %)	0.14

Abbreviations; TA, tubular adenoma.

Supplementary Table S3. Clinicopathological characteristics of colorectal neoplasia resected endoscopically according to age at diagnosis.

	**<40 (n = 159)**	**40-49 (n = 781)**	**50-59 (n = 439)**	**60-69 (n = 630)**	**70-79 (n = 964)**	**≥80 (n = 426)**	***P* value**
Location (6 sites)							<0.001
Cecum, n (%)	11 (6.9 %)	32 (4.1 %)	27 (6.2 %)	51 (8.1 %)	70 (7.3 %)	35 (8.2 %)	
Ascending, n (%)	15 (10 %)	132 (17 %)	105 (24 %)	156(25 %)	272 (28 %)	112 (26 %)	
Transverse, n (%)	31 (20 %)	153 (20 %)	98 (22 %)	164 (26 %)	249 (26 %)	113 (27 %)	
Descending, n (%)	13 (8.2 %)	79 (10 %)	39 (8.9 %)	52 (8.3 %)	90 (9.3 %)	50 (12 %)	
Sigmoid, n (%)	77 (48 %)	299 (38 %)	118 (27 %)	169 (27 %)	207 (22 %)	87 (20 %)	
Rectum, n (%)	12 (7.5 %)	86 (11 %)	52 (12 %)	38 (6.0 %)	76 (7.9 %)	29 (6.8 %)	
Location (3 sites)							<0.001
Proximal, n (%)	57 (36 %)	317 (41 %)	230 (52 %)	371 (59 %)	591 (61 %)	260 (61 %)	
Distal, n (%)	90 (57 %)	378 (48 %)	157 (36 %)	221 (35 %)	297 (31 %)	137 (32 %)	
Rectum, n (%)	12 (7.5 %)	86 (11 %)	52 (12 %)	38 (6.0 %)	76 (7.9 %)	29 (6.8 %)	
Morphology							<0.001
Ip, n (%)	44 (28 %)	188 (24 %)	33 (7.5 %)	54 (8.6 %)	61 (6.3 %)	16 (3.8 %)	
Is or Isp, n (%)	101 (63 %)	502 (64 %)	350 (80 %)	439 (70 %)	702 (73 %)	317 (74 %)	
II, n (%)	14 (8.8 %)	91 (12 %)	56 (13 %)	137 (22 %)	201 (21 %)	93 (22 %)	
Pedunculated type							<0.001
Ip, n (%)	44 (28 %)	188 (24 %)	33 (7.5 %)	54 (8.6 %)	61 (6.3 %)	16 (3.8 %)	
Non-Ip, n (%)	115 (72 %)	593 (76 %)	406 (93 %)	576 (91 %)	903 (94 %)	410 (96 %)	
Lesion size							
<4 mm, n (%)	36 (23 %)	154 (20 %)	129 (29 %)	201(32 %)	263 (27 %)	143 (34 %)	<0.001
5-9 mm, n (%)	74 (46 %)	386 (49 %)	229 (52 %)	288 (46 %)	467 (49 %)	196 (46 %)	
≥10 mm, n (%)	49 (31 %)	241 (31 %)	81 (29 %)	141 (22 %)	234 (24 %)	87 (20 %)	
Histology							<0.001
Carcinoma (pTis, pT1), n (%)	13 (8.2 %)	77 (10 %)	21 (4.8 %)	55 (8.7 %)	82 (8.5 %)	29 (6.8 %)	
High-grade TA, n (%)	33 (21 %)	139 (18 %)	51 (12 %)	72 (11 %)	102 (11 %)	39 (9.2 %)	
Low-grade TA, n (%)	102 (64 %)	543 (69 %)	344 (78 %)	473 (75 %)	732 (76 %)	350 (82 %)	
SSL, TSA, SA, n (%)	11 (6.9 %)	22 (2.8 %)	23 (5.2 %)	30 (4.8 %)	48 (5.0 %)	8 (1.9 %)	
Advanced adenoma, n (%)	61 (38 %)	303 (39 %)	107 (24 %)	173 (28 %)	283 (29 %)	104 (24 %)	<0.001
Endoscopic resection methods							<0.001
Cold polypectomy, n (%)	36 (23 %)	193 (25 %)	243 (55 %)	338 (54 %)	522 (54 %)	258 (61 %)	
Hot snare polypectomy/EMR/ESD, n (%)	123 (77 %)	588 (75 %)	196 (45 %)	292 (46 %)	442 (46 %)	168 (39 %)	

Abbreviations; EMR, Endoscopic mucosal resection; ESD, Endoscopic submucosal dissection; SSL, sessile serrated lesion; TA, tubular adenoma; TSA, traditional serrated adenoma.

Supplementary Table S4. Clinicopathological characteristics of early- vs later-onset colorectal neoplasia detected by screening or surveillance colonoscopy and resected endoscopically.

	**Total (n = 697)**	**Early-onset neoplasia (n = 96)**	**Later-onset neoplasia (n = 601)**	***P* value**
Location (6 sites)				0.011
Cecum, n (%)	43 (6.2%)	3 (3.1%)	40 (6.7%)	
Ascending, n (%)	170 (24%)	11 (11%)	159 (27%)	
Transverse, n (%)	196 (28%)	29 (30%)	167 (28%)	
Descending, n (%)	66 (9.5%)	13 (16%)	53 (8.8%)	
Sigmoid, n (%)	173 (25%)	31 (32%)	142 (24%)	
Rectum, n (%)	49 (7.0%)	9 (9.4%)	40 (6.7%)	
Location (3 sites)				0.012
Proximal, n (%)	409 (59%)	43 (45%)	366 (61%)	
Distal, n (%)	239 (34%)	44 (46%)	195 (32 %)	
Rectum, n (%)	49 (7.0%)	9 (9.4%)	40 (6.7 %)	
Lesion size				0.93
<4 mm, n (%)	298 (43%)	42 (44%)	256 (43%)	
5-9 mm, n (%)	331 (48%)	44 (46%)	287 (48%)	
≥10 mm, n (%)	68 (9.8%)	10 (10%)	58 (9.7%)	
Histology				0.48
Carcinoma (pTis, pT1), n (%)	14 (2.0%)	2 (2.1%)	12 (2.0%)	
high-grade TA, n (%)	53 (7.6%)	10 (10%)	43 (7.2%)	
low-grade TA, n (%)	610 (88%)	83 (87%)	610 (88%)	
serrated adenoma, n (%)	20 (2.9%)	1 (1.0%)	19 (3.2%)	
Advanced neoplasia, n (%)	50 (7.2%)	9 (9.4 %)	41 (6.8 %)	0.39

Abbreviations; TA, tubular adenoma.
